# Polymer Gels: Classification and Recent Developments in Biomedical Applications

**DOI:** 10.3390/gels9020161

**Published:** 2023-02-17

**Authors:** Mariana Chelu, Adina Magdalena Musuc

**Affiliations:** “Ilie Murgulescu” Institute of Physical Chemistry, 202 Spl. Independentei, 060021 Bucharest, Romania

**Keywords:** polymer gels, cross-linked network, hydrogels, biomedical applications

## Abstract

Polymer gels are a valuable class of polymeric materials that have recently attracted significant interest due to the exceptional properties such as versatility, soft-structure, flexibility and stimuli-responsive, biodegradability, and biocompatibility. Based on their properties, polymer gels can be used in a wide range of applications: food industry, agriculture, biomedical, and biosensors. The utilization of polymer gels in different medical and industrial applications requires a better understanding of the formation process, the factors which affect the gel’s stability, and the structure-rheological properties relationship. The present review aims to give an overview of the polymer gels, the classification of polymer gels’ materials to highlight their important features, and the recent development in biomedical applications. Several perspectives on future advancement of polymer hydrogel are offered.

## 1. Introduction

Polymer gels are a versatile, soft, semi-solid class of materials with an intermediate consistency between liquid and solid states. Their cross-linked network can form cavities of different shapes and sizes, in which various molecules and drugs can be trapped [1,2]. The applications of polymer gels are determined by different factors that (i) influence the formation process, (ii) influence the stabilities of gels, and (iii) the relationships between their unique structure and rheological properties. In everyday life, polymer gels are often found in food products (cakes or sausages, ketchup, cheeses), in personal use (cosmetics, shampoos, toothpaste, shaving cream), medical products (tissue engineering, coatings for medical devices, contact lenses, transdermal drug delivery, wound dressing, drug delivery systems), or in various industrial products (adhesives, paints, asphalts), in the sensor industry, and in environmental protection [3,4,5].

In 1894, van Bemmelen introduced the term “hydrogel” [6]. Later, in 1960, Wichterle and Lim described a cross-linked hydrogel based on poly (2-hydroxyethyl methacrylate) (HEMA), which can be applied to the manufacture of contact lenses, drug carriers, and to treat osteoporosis [7]. In 1980, Lim and Sun fabricated a micro capsular membrane composed of cross-linked alginate for use in cell engineering [8]. One year later, a new material for use in wound dressing was developed. This material, based on the natural polymer, collagen, drew attention to the use of polymeric hydrogels for various potential applications [9].

Polymer gels are systems formed by a polymer and a solvent in the arrangement of a three-dimensional (3D) cross-linked polymeric network. Depending on the variation of the external environment, like physical stimuli (temperature, electric and magnetic field, light, pressure) and/or chemical stimuli (pH, ionic strength, molecular species, and solvent composition), the polymer gels can discontinuously and reversibly change their volume [10,11]. Polymeric gels have the capacity to absorb a significant amount of water (tens to hundreds of times greater than the polymer itself) or biological fluids due to the existence of a hydrophilic component [12,13]. The polymer gel can swell until an equilibrium state is established between the osmotic forces and the ability to expand the polymer chains [14,15,16]. The swelling capacity of the functional polymer gels arises from their hydrophilic functional groups that are attached to polymer chains, and from the cross-links between the polymer chains. This leads to a dissolution resistance in polymer gels. Due to a “soft” intermediate state, the polymer gels display a finite shear viscosity [17,18]. This review is focused on analysis of the main characteristics of polymer gels, scrutinizing the results of the latest research, and their biomedical applications as undertaken during the last few years. From Wichterle’s revolutionary work [7] to the newest polymer gel-based developments and tenders, the present review article offers the reader a detailed overview of this area and an outlook regarding further potential developments.

## 2. Classification of Polymer Gels

**Figure 1:**
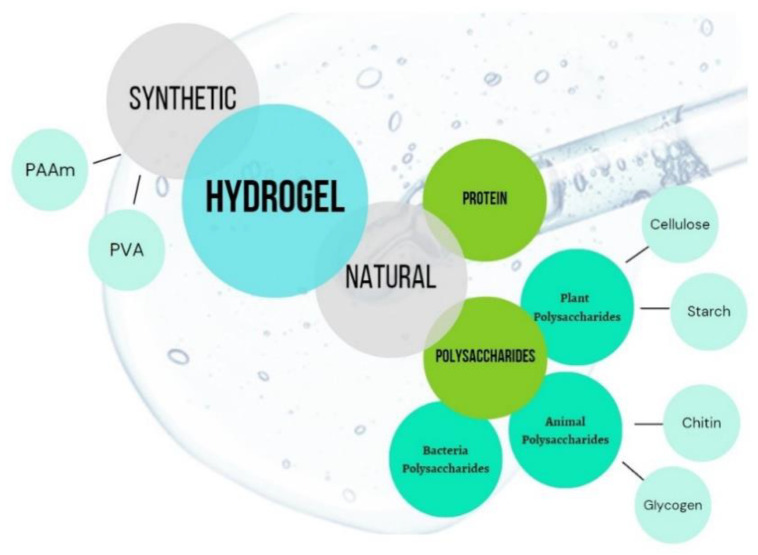
Type of materials used for synthesis of hydrogel based on their origin [15].

Polymer gels can be classified in different categories:

(i) based on their sources: natural or synthetic origin (Figure 1) [15,19].

(ii) based on polymeric composition: homopolymeric, copolymeric, or multipolymeric interpenetrating polymer gels.

(iii) based on type of cross-linking: chemically or physically cross-linked [20].

(iv) based on physical appearance: amorphous (non-crystalline), semicrystalline, or crystalline.

(v) according to network electrical charge: non-ionic (neutral), ionic (anionic or cationic), amphoteric electrolyte (containing both acidic and basic groups), or zwitterionic (containing both anionic and cationic groups in each structural repeating unit).

**Figure 2:**
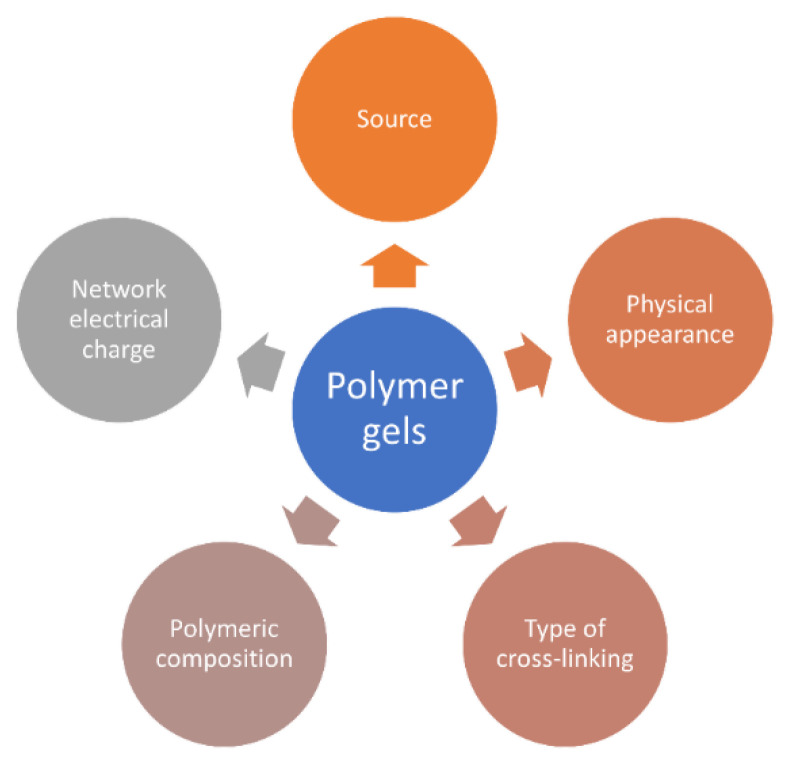
Classification of polymer gels.

In Figure 2 is represented the schematic illustration of the different categories used for polymer gels classification.

### 2.1. Classification Based on the Polymeric Composition

#### 2.1.1. Homopolymeric Polymer Gels

These materials are obtained using a single species of monomer. Depending on the source of the monomer and the polymerization technique, their structure can be skeletal cross-linked. They can be arranged in a block, alternating, or random configuration [21].

#### 2.1.2. Copolymeric Polymer Gels

Copolymeric gels are formed by two or more different monomers that have at least one hydrophilic part [22,23,24,25].

#### 2.1.3. Multipolymer Interpenetrating Polymer Gels (IPN)

This polymer gels type is formed by a cross-linked polymer and a non-cross-linked synthetic and/or natural component polymer [26,27,28].

### 2.2. Classification Based on Type of Cross-Linking

#### 2.2.1. Gels Physically Cross-Linked

Physical gels present multiple advantages as they are easier to be obtained and no cross-linking agents being necessary. They can be formed by a physical (hydrogen) bond, by crystallization, by ionic bonds, by self-assembly of small molecules, and by mechanical dispersion. Physical cross-linking is preferred to chemical cross-linking, when it is possible, to avoid the residual toxicity of chemical additives. Polymer gels can be obtained in the form of aerogels, cryogels, hydrogels, xerogels, nano and microgels, films, or composite materials with micro- and nanoparticles.

The molecular forces that act between the constituents of the “soft” matter depend on the size of the polymer particles and the nature of the medium in which they are dispersed. They include hydrogen bonds, intermolecular associations through van der Walls bonds, hydrophobic interactions, electrostatic interactions, polymer interchain interactions, or local crystallite formation.

Physically cross-linked gels are reversible gels, with temporary bonds between the polymer chains that appear following changes in temperature, pH, or solvent composition. They can be used in different fields including biological applications (biomedicine, drug administration, diagnostic carriers, joint replacement) or for technological applications (food additives, fuel additives, cosmetics, detergents, lubricants, paints) [29,30,31,32,33,34,35].

#### 2.2.2. Gels Chemically Cross-Linked

Chemical gels are formed by the covalent cross-linking of existing polymer chains, which ensures a permanent bond between them [36]. Chemically cross-linked gels are also called irreversible gels. They are usually obtained by four methods:

(i) cross-linking by polymerization, which can be done by addition, condensation, photopolymerization, with free radicals, with electromagnetic radiation, and with plasma.

(ii) polymerization by condensation.

(iii) addition polymerization.

(iv) cross-linking of the polymer chain in random or end-linking processes.

The advantages of polymer gels obtained by addition and condensation are caused by a multifunctional cross-linking agent reacting with the monomer units, thereby initiating the development of the chain. The polymer gels produced in the presence of electromagnetic radiation have the advantage that they can be made at room temperature and physiological pH, even without the addition of a cross-linking agent.

The gels obtained by anionic or cationic polymerization are sensitive to water and, therefore, their use has the disadvantage that they are limited to non-polar monomers, not being able to obtain hydrogels. The degree and type of cross-linking can induce changes in some properties of the network, such as swelling, elasticity, and transport properties [29,37,38,39,40,41].

The types of “bonds” that are formed and the cross-linking methods used establish the physicochemical characteristics of the polymer gels. They have distinct advantages and disadvantages. Table 1 shows the main advantages and disadvantages of physical and chemical cross-linking.

#### 2.2.3. Gels Cross-Linked by Ionizing Radiation

Ionizing radiation is a useful, effective, and clean tool for obtaining polymer gels for biomedical applications [42,43,44,45]. The main advantage of this process is the efficacy of the ionizing radiation at room temperature, and its ability to process any kind of physical material. Using this technique, no residual toxic chemical reagents remain in the final product. Moreover, polymer gels can be sterilized with the cross-linked process at the same time.

### 2.3. Classification Based on the Source of the Used Precursor

#### 2.3.1. Synthetic Gels

Synthetic gels show adaptable mechanical and degradation characteristics and have multiple applications in engineering and materials science [46,47,48]. This type of polymer is often used in regenerative medicine, bioprinting, energy storage, or drug delivery. Some of the most common polymer gels are based on PEG (poly(ethylene glycol)) [49], PVA (poly(vinyl alcohol)) [50], PMMA (poly(methyl methacrylate)), PHEMA (poly(hydroxyethylmethyl acrylate)) [51,52], polyurethanes, poly(amino acids), and PVP (poly(vinyl pyrrolidone)) [53].

Synthetic polymer gels are specifically designed to imitate biopolymers from organic forms. They have been configured and developed with well-designed functions for various applications, including industrial ones. Thus, both “stimuli-sensitive” and “environmentally sensitive polymers” can be found. They could have the ability to respond to insignificant changes in the environment. The capability of these polymers can be seen in both the rapid changes of their structure and in the reversibility of the phase transitions. These transitions can be of different types, like sol-gel transitions, changes in solubility, shape, surface properties, or the formation of complex gels [54,55].

#### 2.3.2. Natural Polymer Gels

Natural polymer gels contain biopolymers like polysaccharides (xanthan gum, alginate, starch, chitosan), proteins (fibrin, collagen, or gelatin) or polynucleotides, that are found in living organic systems as main components [56,57,58,59]. Bio-based polymer gels have unique and diverse characteristics, the most important being the biodegradability and biocompatibility compared to synthetic hydrogels. Due to their versatile properties, these polymeric gels have led to a significant and growing interest in the field, due to their connection with the natural environment and new and attractive functionalities.

To make gels with different shapes and structures, several types of gelators with different molecular weights can be combined to obtain supramolecular hybrid hydrogels [60].

The mixture between natural and synthetic polymers leads to hybrid hydrogels. These are functionalized materials with unique attributes capable of incorporating the benefits of both types of included polymers, like biodegradability, a good control of rigidity, viscosity, and high strength [61,62].

**Figure 3:**
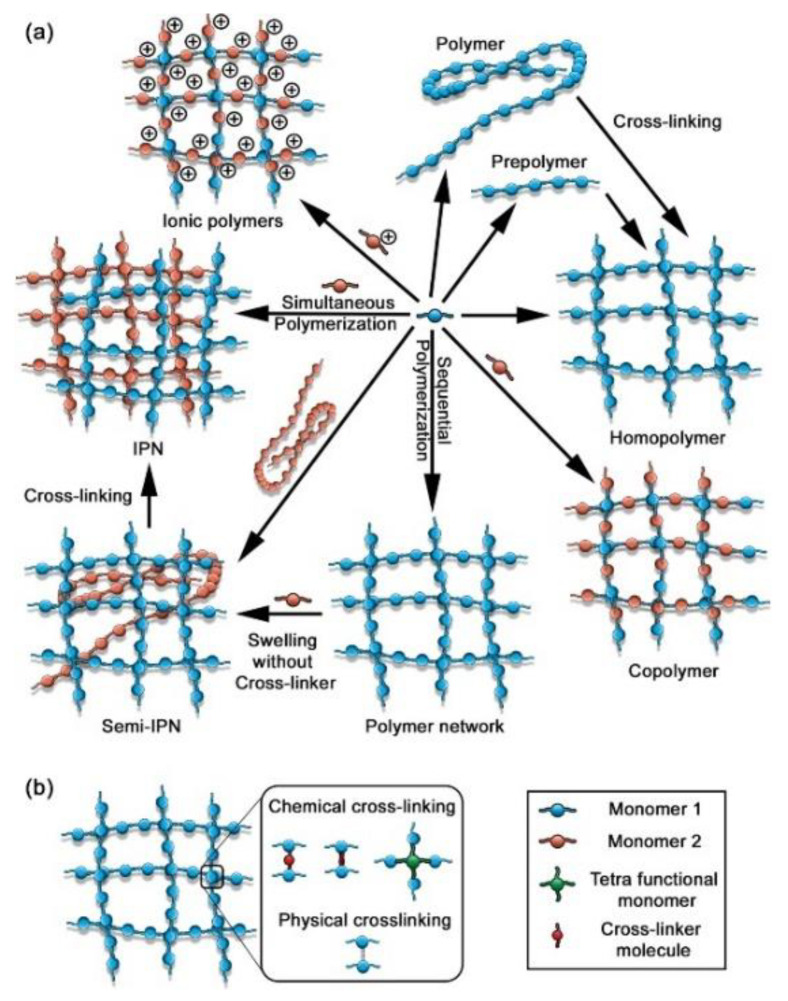
(**a**) Representation of various categories of hydrogels. (**b**) Preparation by cross-linking between polymer chains [63].

A schematic representation of various type of polymeric hydrogels is illustrated in Figure 3 [63].

## 3. Type of Stimuli-Responsive Polymer Hydrogels

**Figure 4:**
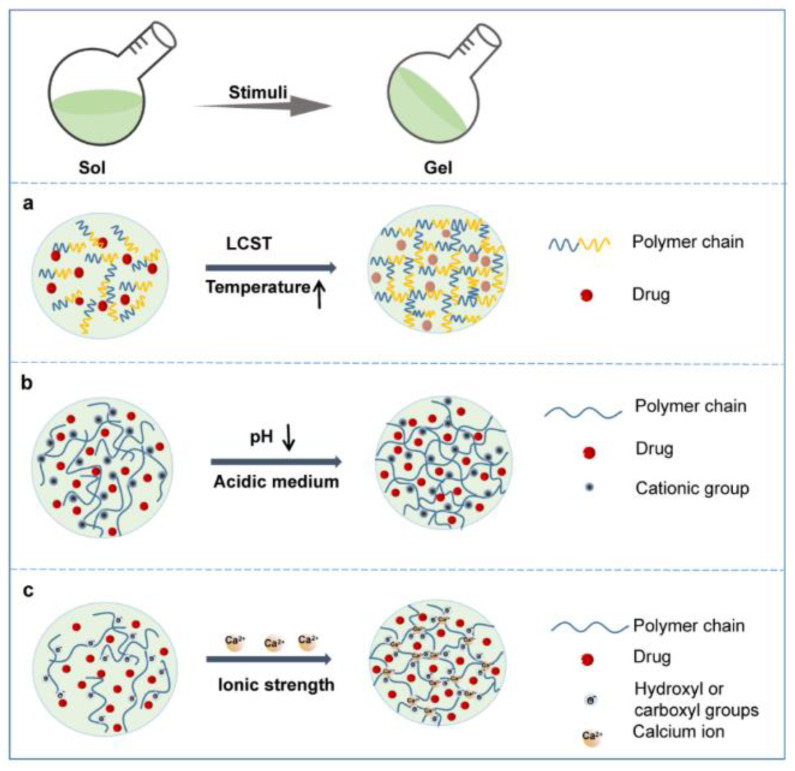
A graphical representation of polymer gels phase transition of representative hydrogels. (**a**) temperature responsive; (**b**) pH responsive; (**c**) tonic strength responsive [64].

Polymer hydrogel can be considered a smart material in relation to multiple application. It can be synthesized to respond to different stimuli in the human body, like ionic strength, pH, and/or temperature. These triggered mechanisms can be used to release drugs or bioactive compounds. A schematic representation of phase transition of a polymer hydrogels in response to different stimuli is given in Figure 4 [64].

### 3.1. Thermoresponsive Polymer Hydrogels

It was demonstrated that a small change in temperature can affect the equilibrium between hydrophobic and hydrophilic polymer segments by causing a sol-gel phase transition [65]. These kinds of polymer hydrogels are a category of the supramolecular hydrogels that are transformed in gels through hydrophobic interactions. Due to the property to form gel at higher temperatures and to return to a liquid state at lower temperatures, they can be used as biocompatible injectable thermogels [66].

Lee et al. [67] demonstrated that the administration by subcutaneous injection of human C-peptide conjugated with an elastin-like biopolymer (K9-C-Peptide) develops a hydrogel depot that can slowly release the human C-protein into the circulatory system over 19 days. It was demonstrated the long-term influence on hyperglycemia-induced vascular dysfunction by applying an aortic endothelium prototype in diabetic mice.

Dong et al. [68] synthesized an injectable thermo-sensitive chitosan hydrogel that has been incorporated into a 3D-printed poly(ε-caprolactone) (PCL)-based scaffold in order to create a hybrid scaffold. The obtained materials maintained durable compressive strength and provided a promising micro-environment useful for cell growth and in osteogenesis. The 3D scaffold can be further used as a possible bone defect repair for in vivo and subchondral applications.

Cao et al. [69] reported the first development of a transdermal hydrogel made of 5-aminolevulinic acid for use in photodynamic therapy for skin disease. Two triblock copolymers–poly(d,l-lactide-*co*-glycolide)-*b*-poly(ethylene glycol)-*b*-poly(d,l-lactide-*co*-glycolide) of different block lengths are produced by changing the blending ratio and using both sol–gel transition and gel–sol transition of a thermogelling arrangement. This research has proved that the formation of the “block blend” biomaterials is possible and this suggests further development of more intelligent drug delivery systems.

### 3.2. pH-Responsive Polymer Hydrogels

Polymer hydrogels, which are pH-responsive, are a group of biomaterials that were bonded with polymer chain acidic or basic groups, and which demonstrate suitable physical or/and chemical properties in a certain pH domain. The importance of pH-responsive polymer hydrogels is due to their swelling reaction in response to the pH of various body organs, the digestive system, or fluids [70,71,72]. The variations of pH in different parts of the digestive system are very important. In this regard, the pH-sensitive profile of the marine polysaccharide fucoidan–chitosan (FUC-CS) system prevents degradation under acidic gastric conditions and ensures an efficient drug absorption in the intestine [72,73].

Suhail et al. have developed pH-responsive hydrogels of carbopol, chondroitin sulphate, and polyvinyl alcohol using the free radical polymerization method with acrylic acid in the presence of ammonium persulphate and ethylene glycol dimethylacrylate for oral controlled drug delivery [74]. They demonstrated the capability of the developed pH-sensitive polymer hydrogel to obtain maximum swelling and drug release at two pH values (4.6 and 7.4, respectively). No cytotoxic effect was observed on human cancer cells in the colon. The pH-responsive hydrogels have the property to protect the stomach from harmful drug side effects and to preserve the drug from the acidic medium in the stomach.

Schoener et al. [75] have developed a pH-responsive polymer hydrogel based on poly(methacrylic-grafted-ethylene glycol) with various amounts of hydrophobic PMMA nanoparticles. They demonstrated the ability of the hydrogel to hold different amounts of doxorubicin, and to locally release doxorubicin in the colon for use in the treatment of colon cancers. Low pH was applied to simulate the pH of the stomach and then altered to neutral conditions to mimic the upper small intestine. No cytotoxic effect was detected using gastrointestinal tract and colon cancer cell lines, from either varying concentration or times of exposure.

Another study demonstrated that a developed pH-responsive hydrogel using acrylamide and methyl acrylic acid in the presence of N-N′-methylene bisacrylamide by free radical polymerization could be used as a useful oral site-specific release platform to deliver gastric-sensitive bioactive material to the small intestine route [76].

**Figure 5:**
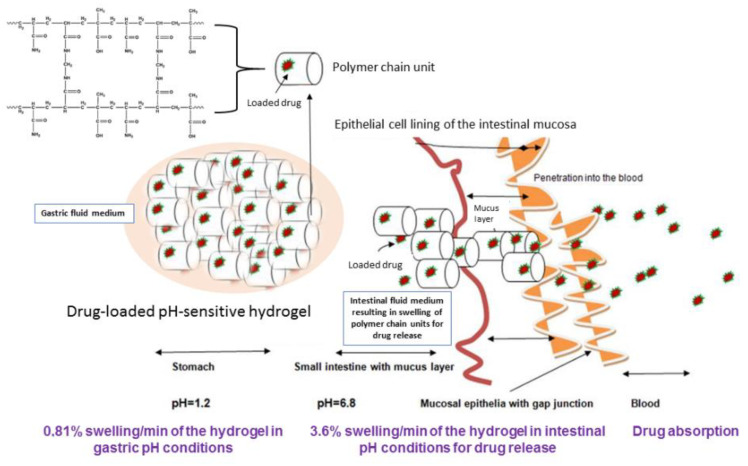
Mechanism of pH-responsive hydrogel system [76].

In Figure 5, the schematic representation of the pH-responsive polymer hydrogel, ascribed to a minimum swelling ability in a simulated gastric pH, and a significant swelling and release of theophylline as drug in the simulated intestinal pH for absorption of the loaded prototype drug, is shown.

### 3.3. Light and Chemical-Responsive Polymer Hydrogels

Light-responsive polymer gels are promising biomaterials with potential for use as sensors [77] or for drug delivery [78], based on their activation by light.

Anugrah et al. [79] has developed a near-infrared-responsive polymer hydrogel based on alginate cross-linked with tetrazine via the Diels–Alder reaction as a controllable drug carrier. A near-infrared sensitive indocyanine green and doxorubicin were included in the polymer hydrogel matrix through gelation. The obtained hydrogels demonstrated a controlled release profile under simulated physiological conditions and a rapid release profile of doxorubicin under near-infrared irradiation. The near-infrared-light promoted the generation of reactive oxygen species caused by indocyanine green, which subsequently released the entrapped doxorubicin.

Wang et al. [80] has succesfuly incorporated a fluorescent carbon nanoparticle into poly(*N*-isopropylacrylamide-*co*-acrylamide) nanogels via the one-pot precipitation copolymerization method. The resultant hybrid nanogels can bring together properties for cell imaging, fluorescent temperature sensing, and near-infrared responsiveness for drug delivery systems by accelerating the drug release and by enhancing its therapeutic efficiency. Subjected to laser excitation, it was proved to light up melanoma B16F10 cells from a mouse.

## 4. Structure-Effect Relationship

**Figure 6:**
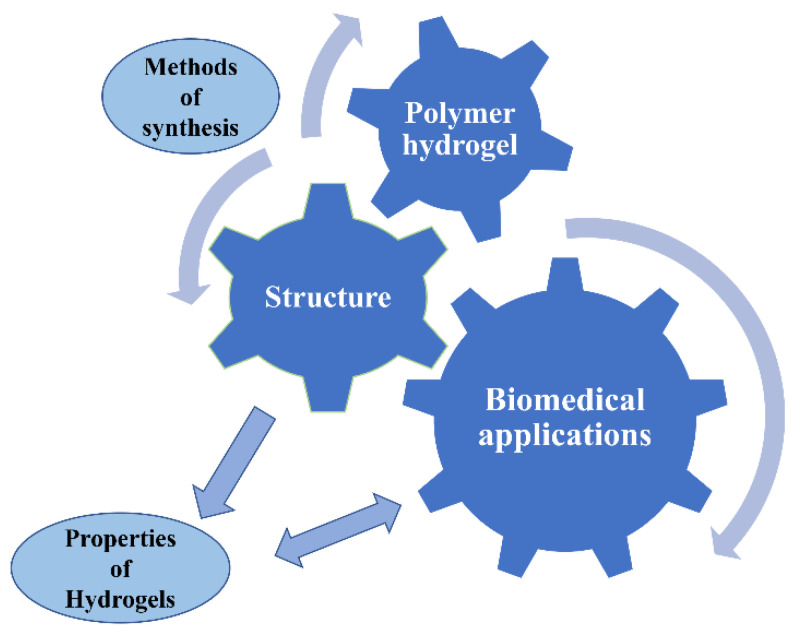
Schematic representation of structure-effect relationship of polymer gels.

The assessment of the relationship between the structure and its effect is extremely important for selection of specific applications (Figure 6). The swelling behaviour and mechanical properties are very important when determining a relationship between these properties and the structural parameters of polymer hydrogels.

### 4.1. Mechanical Properties

The cross-linked density and the water content determine the mechanical properties of a polymer gel. The mechanical properties of polymer gels are determined using rheological analysis used to measure the viscoelastic properties. When stress is applied to a sample, the response under deformation is measured.

A dermal filler hydrogel was synthesized using hyaluronic acid cross-linked with polyethylene glycol diglycidyl ether and containing calcium hydroxyapatite, glycine, and proline [60]. The physical-chemical characterization was carried out by measuring G′ (elastic modulus), G″ (viscous modulus), and tan δ (phase angle tangent). This showed similar trends under different thermal conditions. The results showed that the product was not affected by storage conditions. It highlighted that the material has a pseudoplastic behaviour (non-Newtonian shear thinning) and that the viscosity of the dermal filler decreased with the increase of the shear rate, under all the conditions in which it was tested [60]. The characteristics of this hyaluronic acid hydrogel make it recommendable for use in the cosmetic industry as a filling material for facial rejuvenation by forced injection through a needle into the tissues.

**Figure 7:**
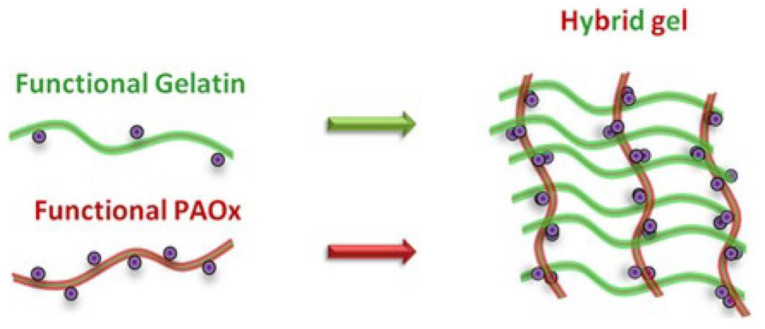
Schematic representation of the formation of a hybrid hydrogel [81].

A hybrid hydrogel was made using a mixture of synthetic and natural polymer building blocks (gelatin and PAOx used as precursor materials). These polymers were chosen to use a combination of the mechanical stability of synthetic polymers with the cell-interactive properties of natural polymers [81]. Thiolated gelatin (SH-gel) was prepared and a hybrid hydrogel obtained using thiolene radical crosslinking, which ensured the interconnectivity of PAOx and gelatin precursors. Figure 7 shows the formation of the hybrid hydrogel composed by gelatin and functionalized with thiol and PAOx, which have been functionalized with alkene. The obtained material showed an increased mechanical stability as well as a thermosensitive behaviour at temperature variations around 30 °C [81].

### 4.2. Polymer Hydrogel Swelling Properties

The swelling behaviour of biopolymer hydrogels can be described using simulated biological fluids. The swelling properties depend on pH, ionic strength, and temperature [82]. The free and bounded water in relation to the total water content indicated the swelling properties. The water content can be established using thermal analysis methods. By knowing the swelling properties, the degree of crosslinking, the mechanical properties, and the rate of degradation can be calculated.

### 4.3. Porosity

Porosity has a significant role in the applications of polymer hydrogels. The swelling and release rate of drugs are influenced by the network porosity of polymer hydrogels [83]. There are several techniques that can be used to assess the polymer gels porosity, like gas absorption, optical microscopy, scanning electron microscopy, transmission electron microscopy, atomic force microscopy, and capillary flow porosity.

The high porosity of the polymer hydrogel made it very permeable to various types of drugs, making it suitable for drug delivery in controlled conditions [84,85,86,87,88,89]. In a drug delivery study, the polymer gel’s ability to release drugs in a sustained manner for a long period represents a considerable advantage, following an increase in a drug’s concentration over a long time. Both physical and chemical methodologies can be employed to increase the affinity between the polymer hydrogel matrix and the drug, and to extend the release time [90,91].

## 5. Applications of Polymer Gels

**Figure 8:**
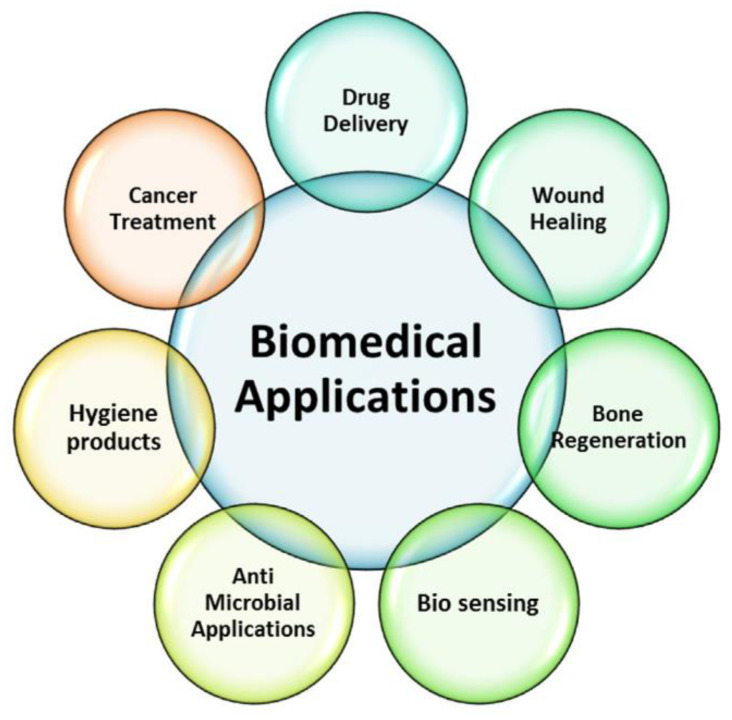
Schematic representation of biomedical applications of polymer gels [92].

Polymer hydrogels possess a broad range of applications due to their distinctive patterns and their capability to be applied and to function in various environments. As a result of the water content of polymer hydrogels, they are sufficiently adaptable for use in a large variety of industrial, pharmaceutical, and biological applications. In Figure 8, some of the biomedical applications of polymer hydrogels are shown [92].

A summary of different categories of polymer gels cross-linked by physical, chemical, or irradiation methods used in biomedical applications is presented in Table 2.

### 5.1. Drug Delivery

In order to use polymer hydrogels as drug delivery systems, they must have the following properties: (i) a porous structure [113]; (ii) an adequate release rate [114]; (iii) the ability to protect the drug [114], and (iv) biodegradable and biocompatible [115,116,117].

For transdermal drug delivery, novel, non-aqueous, directly-compressed tablets containing drugs formulated from common solid pharmaceutical tablet excipients have been developed for use with arrays of microneedle patches in hydrogel form. These patches were prepared and tested for in vivo delivery of amoxicillin, levodopa, and levofloxacin at therapeutically significant concentrations in rats [118].

Using chitosan as a natural polymer and polyurethane containing azomethine as a synthetic polymer, biodegradable hybrid hydrogels were developed for controlled release applications of drugs like 5-fluorouracil. These hydrogels showed good drug release behaviours of 50% of 5-fluorouracil, proving themselves able to be used for this purpose [119].

For the delivery of a drug with antioxidant and anti-inflammatory properties, an injectable hydrogel depot and tissue adhesive loaded with epigallocatechin-3-gallate was prepared. It showed a good response in the sustained release of the drug and could be used as a promising treatment in tissue degeneration to improve inflammatory disorders. [120].

A new smart hydrogel was developed as a sustained release material in the form of a compressed tablet, based on natural polysaccharides isolated from the seeds of *Salvia spinosa*. The sustained release potential of this hydrogel was investigated for its pH-dependent and salt-sensitive swelling in two steps, both before and after the tablet was prepared in tablet form. The study on the controlled release of theophylline (<80%) from the seeds of *Salvia spinosa* was monitored at the pH of the gastrointestinal tract. This pH-sensitive material showed good potential for sustained and targeted drug delivery [121].

Polymer hydrogels are ideal materials to adsorb and store different types of drugs to release them in a predetermined way for a fixed period [122].

For colorectal cancer therapy, guar-chitosan dialdehyde-based hydrogels cross-linked in situ for dual drug release were synthesized. These aimed at both simultaneous chemotherapy and pain relief in colorectal cancer therapy. Hydrogels based on guar gum and chitosan-dialdehyde cross-linked in situ were prepared for controlled dual release of curcumin and aspirin. The hydrogels protected the drugs against absorption in the stomach and small intestine, showing potential as a combined therapy for colorectal cancer [123].

### 5.2. Wound Healing

**Figure 9:**
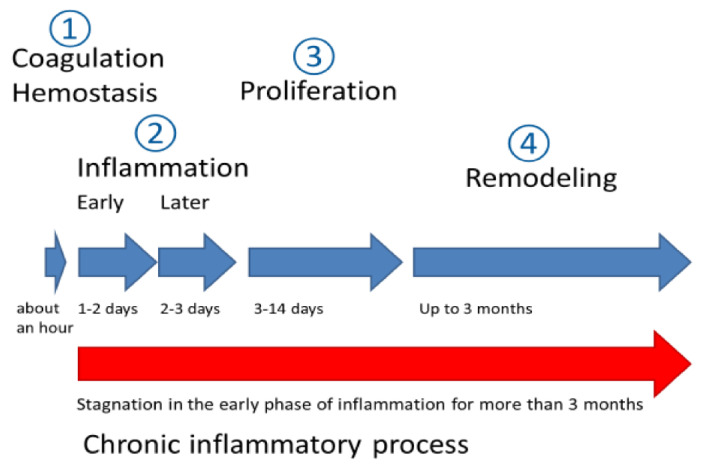
Stages of wound healing process [124].

The wound healing process comprises four stages, shown in Figure 9 [124].

In order to be efficient and to have the best performance, an ideal wound dressing requires several properties: (i) to protect the wound from infection caused by microorganisms; (ii) to be biocompatible; (iii) to have the capacity of moisture retention; (iv) to have a gas permeability; and (v) to provide a moist environment in order to reduce the formation of scar [125].

Ying et al. obtained an extracellular matrix mimic hydrogel containing collagen I and hyaluronic acid by covalent cross-linking of hyaluronic-acid-tyramine (HA-Tyr) through horseradish peroxidase and H_2_O_2_ for use as effective wound dressing [126]. The prepared hydrogel has the capacity of autonomous healing promotion by growing vascular cells and then encouraging the closure of wound.

Ding et al. developed a collagen, chitosan, and dialdehyde-terminated polyethylene glycol self-healing polymer gel based on dynamic imine bonds for wound dressing [127]. The obtained hydrogels have shown good healing capacity, thermal stability, antibacterial activity, exceptional hemostatic ability, and injectability.

Yu et al. [128] developed an injectable hydrogel of carboxymethyl chitosan with γ-polyglutamic acid and polydopamine hydrogel for antibacterial applications and the avoidance of tumor recurrence. The carboxymethyl chitosan-based hydrogel showed a good biocompatibility.

### 5.3. Bone Regeneration

Bone infections, trauma, or bone diseases caused by aging, fractures, cartilage damage, or bone defects, including osteoarthritis, substantially affect people’s quality of life. Bioactive materials-based hydrogels can stimulate bone regeneration by acting as a bionic extracellular matrix. Recently, biomimetic polymer hydrogel materials have gained attention in bone repair as they facilitate adhesion, proliferation, and differentiation of stem cells [129,130]. An important role in sustaining the balance of the mineral supply in the organism is played by mineral ions like copper, magnesium, calcium, and zinc. These metal ions are linked to the polymer chains in order to form efficient polymer hydrogels which can accelerate the regeneration of the bone.

A macroporous GelMA-structured hydrogel obtained by incorporating MgO nanoparticles based on thiol-ene click reactions was developed [131]. These hydrogels presented good mechanical properties and a porous structure. In vivo experiments revealed an extracellular matrix microenvironment for enhancing the osteogenic differentiation to promote bone tissue regeneration.

### 5.4. Cancer Treatment

As a worldwide health problem, cancer has become the primarily cause of mortality. The treatment of cancer in different stages is currently done using surgery, radiotherapy, chemotherapy, immunotherapy, and targeted molecular therapy [64]. Chemotherapy is the classic treatment and is usually used in the targeted treatment of certain types of tumors or types of cancer. The main disadvantages are the side effects (which are severe, most of the time) and the low specificity of many antitumor drugs that fail to induce the selective death of tumor cells. Due to the side effects associated with the high cytotoxicity of chemotherapeutic compounds, a significant interest has focused on the design and manufacture of a new effective system for cancer treatment. Among numerous other formulations, like films, suspensions etc., polymer hydrogels represent the most adequate drug delivery systems in cancer therapy. Temperature, pH, and ionic responsive hydrogels are efficient as drug release systems. In response to various stimuli like temperature, pH, light, magnetic, or electric fields, or enzymes, drugs are released from the smart polymer gels. In response to the side effects of anticancer therapies, sustained studies have been carried out in recent years to reduce the amount of their cardiotoxicity. Thus, to improve their biocompatibility, as well as their efficiency, significant effort has been made to develop new delivery systems as alternatives to classic cytotoxic anticancer drugs [132].

**Figure 10:**
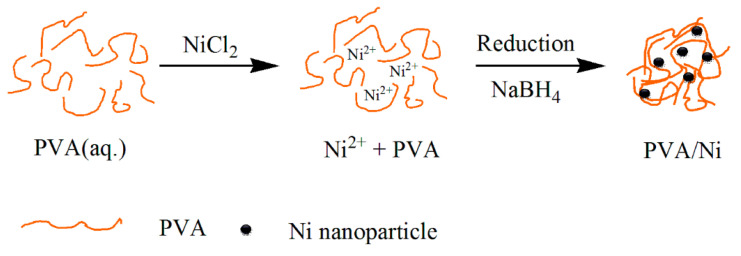
The schematic representation of simultaneous formation of the magnetic gel containing Ni nanoparticles [133].

A new type of magnetic PVA gel containing nickel nanoparticles was developed through a simple one-step procedure (Figure 10) [133].

**Figure 11:**
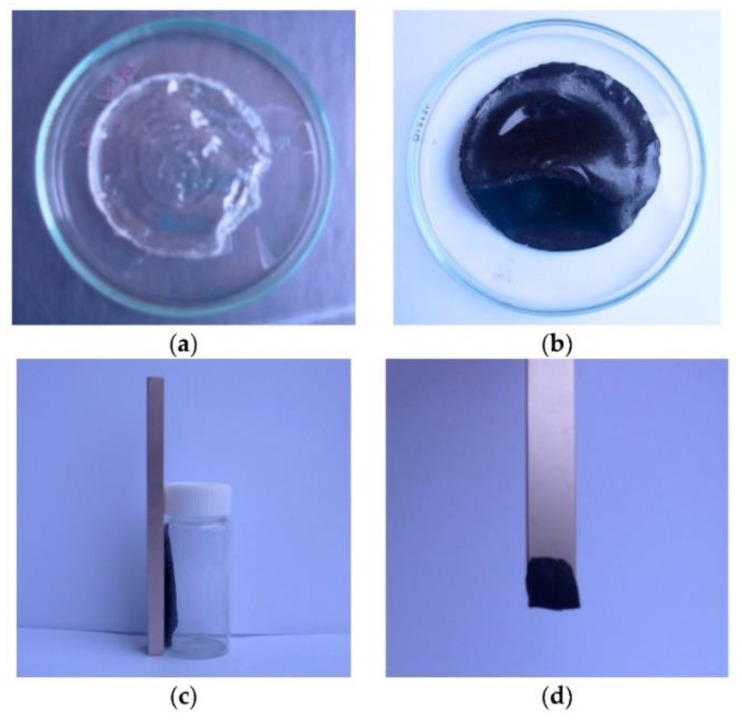
Images of the synthesized material (**a**) PVA gel; (**b**) PVA/Ni magnetic gel; (**c**) Ni-nanoparticles (NPs) in the magnetic field; (**d**) PVA/Ni magnetic gel in the magnetic field [133].

The obtained material could be used in anti-cancer drug delivery and biotechnology (Figure 11).

Gao et al. [134] developed a hybrid, injectable, thermosensitive hydrogel system for the simultaneous delivery of co-encapsulated norcantharidin nanoparticles and doxorubicin via intratumoral administration for hepatocellular carcinoma. The in vivo testing on a mice tumor model, inhibited tumor growth and angiogenesis. Recently, different thermosensitive hydrogels for localized cancer therapy were developed, based on Pluronic F127 with titanium carbide [135], poly(d,l-lactide)-poly(ethylene glycol)-poly(d,l-lactide) with indocyanine green [136], chitosan and silk sericin with tegafur - protoporphyrin heterodimers [137], chitosan and hyaluronic acid with indocyanine green, imiquimod, and cyclophosphamide [138], methylcellulose with IR820 [139], and Pluronic F127 with black phosphate nanosheets and docetaxel [140].

### 5.5. Hygiene Products

Recently, numerous studies have been conducted on the incorporation of natural polymers like cellulose, starch, alginate, and xanthan gum to produce natural, biodegradable, non-toxic, and biocompatible superabsorbent materials [141,142,143].

As a superabsorbent biopolymer, chitosan has been intensively studied for the cosmetic industry in the manufacture and production of various sanitary products for women and children [144,145,146].

Dry hydrogels in the form of covalently cross-linked sodium carboxymethyl cellulose and hydroxyethyl cellulose films were synthesized, employing citric acid as a cross-linking agent. The films showed excellent water absorption and can be targeted as absorbent materials for personal care [147].

Two new superabsorbent hydrogels based on carboxymethyl guar cross-linked with bentonite borax and fumed silica particle reinforcement were synthesized. The incorporation of silica particles showed a positive effect on water absorption capacity, showing that the hydrogels can be used as disposable hygiene products [148].

In recent years, significant attention was focused on the production and characterization of the stability, formulation, and antimicrobial assessment of hand sanitizers to ensure their stability and efficiency.

The gel formulation as hand sanitizer must fulfil several requirements: (i) to avoid the risk of leakage; (ii) a pleasant smell; (iii) fast absorption; (iv) no reduction of the rate of alcohol (the optimal content being from 60% to 90%) [149].

Due to the significant biocompatibility of cellulose, its use as cellulose-based hydrogel is widely studied [150,151,152]. A gel hand sanitizer was manufactured utilizing silver nanoparticles as the antimicrobial agent and coated with chitosan in different concentrations as the stabilizing agent [153].

### 5.6. Antimicrobial Applications

Severe wound infections are one of the primary factors that cause disease, disability, and even death. To avoid infections, modern treatment is dependent on antimicrobial drugs like antibiotics, that can act to destroy the pathogens or to inhibit their growth. Antibiotic treatment frequently proves to be ineffective in destroying infections in chronic non-healing wounds due to the development of multiresistant microbes [154,155]. Antimicrobial wound dressings have drawn wide-ranging attention in the last few years.

Gupta et al. reported the fabrication of silver nanoparticles loaded in a biosynthetic bacterial cellulose hydrogel to develop a wound hydrogel dressing [156]. The nanoparticles of silver were obtained by a green procedure using an aqueous solution of curcumin and hydroxypropyl-β-cyclodextrin. The obtained biocompatible hydrogel dressings demonstrated a broad spectrum of antimicrobial activity against three pathogenic microbes that usually infect wounds: *Pseudomonas aeruginosa*, *Staphylococcus aureus*, and *Candida auris*. An injectable hydrogel based on poly(vinyl alcohol), silk sericin loaded with microspheres of poly(vinyl alcohol) containing gentamicin, vancomycin, and their combination was developed to accelerate the healing of burn wounds and for infection prevention. The synthesis method was based on inverse emulsion cross-linking [157]. The results have shown the synergistic antimicrobial effects of gentamicin and vancomycin against *Staphylococcus aureus*, *Pseudomonas aeruginosa*, and *Escherichia coli*. In vivo studies on a rat burn model showed a cell migration and collagen deposition that promoted the early re-epithelialization and burn wound healing.

### 5.7. Bio-Sensing Applications

Polymer hydrogel-based biosensors have received significant attention in recent years since they are extremely sensitive, easy to fabricate, and can be applied in a range of fields (detection of drugs, diagnosis of many diseases, and environmental domain for detecting the aqueous contaminants).

Their use in the biomedical domain is due to their resemblance to biological tissue and their biocompatibility.

The preparation of hydrogels in the form of porous cell-like films incorporating Prussian blue nanoparticles and various enzymes on electrode surfaces for electrochemical biosensing has been reported. They were made by drop-casting composite hydrogels on the surface of SPE-type screen-printed flexible electrodes. The two amperometric biosensors developed based on hydrogel composites integrating glucose oxidase and alcohol dehydrogenase showed a high sensitivity for the rapid detection of glucose and ethanol in serum. Hydrogels can be considered electrochemical biosensing platforms due to the possibility of the efficient immobilization of some enzymes and nanomaterials in their matrix for the detection of a variety of analytes [158].

A new enzymatic biosensor was prepared for the detection of trimethylamine N-oxide (TMOA) which contains three enzymes: trimethylamine N-oxide-reductase, glucose oxidase, and catalase. It presented a signal linearly dependent on the TMOA concentration in a range between 2 µM and 15 mM, with the lowest detectable concentration being 10 µM TMOA [159].

A new additive manufacturing strategy to efficiently produce layered gelatin hydrogel microfibers bonded to 3D printed thermoplastic structures of various shapes has been developed. This can be applied to optimize the preparation and 3D modeling of electrospun hydrogel fibers using cross-linked hydrophilic polymers and oligomers, for various cell cultures or bio-sensing applications [160].

Different electrochemical biosensors for monitoring microbial metabolites and biomarkers for different types of human microbiome, especially on the gastrointestinal microbiome, have been studied to improve the diagnosis and monitoring of diseases such as gastrointestinal diseases [161].

A new hybrid hydrogel material was developed using mechanochemical incorporation of 3-((7-hydroxy-4-methylcoumarin)methylene)aminophenylboronic acid into the aragose matrix. This hydrogel can selectively and rapidly detect biogenic polyamines spermine and spermidine using a fluorescence turn-on method. The obtained smart platform can be further used to measure the spermine levels in blood plasma and human urine [162].

Wang et al. [163] have produced a new, highly-sensitive, and cost-effective DNA hydrogel sensor for visually (with the naked eye) quantitative detection of miRNAs with potential uses in nucleic acid biosensing. An enzyme polysaccharide hydrogel was designed and manufactured to be targeted by β-mannosidase for delivering bovine serum albumin and lysozyme when it is subjected to β-mannosidase in vivo [164].

**Figure 12:**
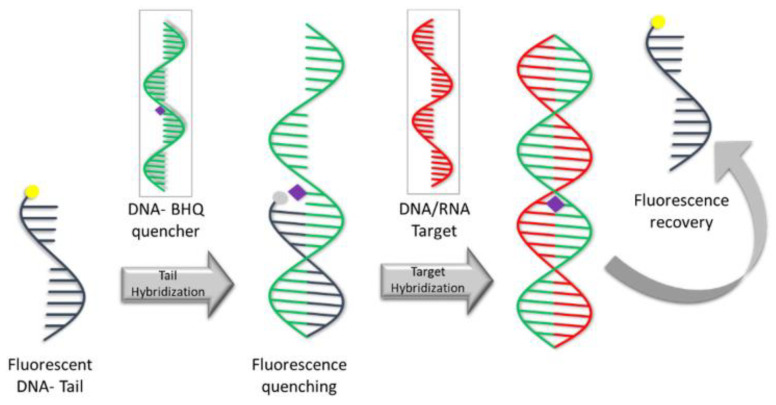
The mechanism of DNA-PEG hydrogels detection assay [165].

An important role in specific biomolecule detection is provided by the three-dimensional (3D) polymer hydrogel network structure. The cross-linked functionalization of DNA with PEG can be used for the detection of short oligonucleotides in complex media [165]. The scheme of the detection assay is presented in Figure 12.

Several protease matrix metalloproteinase responsive hydrogels have been explored as possible protein-responsive drug delivery approaches [166,167], as cancer biomarkers, for the measurement of protein biomarkers [168,169,170,171], and as enzyme-responsive polymer hydrogel for rapid detection of strains such as *Escherichia coli*, *Staphylococcus aureus*, and *Pseudomonas aeruginosa* [172,173,174,175].

Table 3 illustrates some of the latest polymer hydrogels used in possible applications.

## 6. Conclusions and Future Perspectives

The present review aims to provide an overview of polymer hydrogels, their classification depending on different categories, and the possible applications based on their cross-linking. The biomedical applications of polymer hydrogels are also discussed. 

In summary, polymer hydrogels represent a large system made of small monomers physically or chemically cross-linked. The most representative properties, like biocompatibility and biodegradability, make them suitable for pharmaceutical, biological, biomedical, food industry, and environmental applications.

A future development can be seen in the inclusion of various nanomaterials into the polymer hydrogels matrix for use in specific target applications. This possibility can be applied to develop new stimuli-sensitive hydrogels responsive to like light, temperature, pH, and electric field.

The rapid development of the science of polymer gels is a consequence of the importance of these materials and their application in both material and biomedical domains. Nevertheless, there are still various challenging problems that must be addressed in order to fully understand their properties from a more fundamental point of view.

Due to their functional versatility that offers the possibility of their application in many differing fields, including some high-tech disciplines that present a potential for future development, polymer gels deserve sustained efforts towards future study and development.

## Figures and Tables

**Table 1:** Advantages and disadvantages of physical and chemical cross-linking.

Type of Cross-Linking	Advantages of Polymer Gels	Disadvantages of Polymer Gels
Physical cross-linking	➢ is a homogenous reversible gel. ➢ is formed by molecular entanglements, H-bonding, ionic, or hydrophobic forces. ➢ it can be dissolved by changing the environmental conditions (ionic strength, pH, temperature). ➢ the gelation process occurs under mild conditions. ➢ absence of any chemical cross-linking agents. ➢ the preparation method does not use chemical modification. ➢ is less toxic. ➢ led to inconsistent in vivo performance. ➢ the presence of hydrophilic and hydrophobic areas. ➢ can easily incorporate bioactive molecules.	➢ lower bond energy. ➢ lower cross-linking degree. ➢ weak viscoelastic properties. ➢ weak bonds. ➢ less stable against degradation. ➢ poor mechanical properties.
Chemical cross-linking	➢ is a non-homogeneous permanent or irreversible gel. ➢ covalent cross-linking bonds. ➢ it may be charged or non-charged depending on the nature of functional groups from their structure. The charged polymer gels (i) generally reveal changes in swelling at pH variation, or (ii) they can suffer changes in shape when it is subjected to an electric field. ➢ forms strong polymer gel bonds. ➢ satisfactory viscoelastic properties. ➢ an increased resistance to degradation. ➢ it is prepared using chemical modification. ➢ it is flexible to dissolution, degradation, and chemical modification. ➢ better mechanical properties. ➢ it can be used in cosmetics, pharmaceuticals, medicine, food industry, and agriculture.	➢ the presence of toxic agents in the synthesis process. ➢ it must be washed in order to remove the residue.

**Table 2:** Applications of polymer gels cross-linked by physical, chemical or irradiation methods.

Type of Polymer Gels	Gels Physically Cross-Linked	Gels Chemically Cross-Linked	Gels Cross-Linked by Irradiation	Applications	References
Methylcellulose hydrogel	yes	_	_	Thermoresponsive materials	[93]
Nanocomposite hydrogel materials (cellulose polymers and biodegradable nanoparticles)	yes	_	_	3D printing, adhesives, injectable biomaterials, and foods	[94]
iota-carrageenan (C_i_) phenylboronic acid functionalized hydroxylpropylmethyacrylate copolymer (PBA)-based (C_i_-PBA) gel	yes	_	_	Gels for contraception	[95]
Hyaluronic acid (HA) cross-linked using DVS or BDDE, alone or in combination with fibrin	yes	_	_	In vivo remodelling processes	[96]
Gel platform based on poly(ethylene glycol) (PEG) with poly(hydroxyethyl methacrylate-acrylic co-acid)	yes	_	_	Multi-functional gel for wearable electronics, soft actuators, and robotics (inclusive 3D-printing)	[97]
polyvinyl alcohol (PVA), acrylic acid (AA), ammonium persulfate (APS) and Fe^3+^	yes	_	_	High-performance strain sensors	[98]
Xanthan hydrogels with both alkaline and acid solutions as new solid electrolytes	yes	_	_	High-conductivity solid electrolytes for Al-air primary cells	[99]
Highly viscously thiol-modified cross-linked hyaluronate (TCHA)	-_	yes	_	Clinical field (Vitreous Body Substitute)	[100]
Poly(N-isopropylacrylamide) (PNIPPAm) gel with ethanol	_	yes	_	polymeric gel storage for liquid fuels	[101]
Highly carboxylated cellulose nanofibril (CNF) cryogel beads using maleic anhydride (MA)	_	yes	_	Heavy metal ions (Cu (II)) removal	[102]
Maleimide-modified c-polyglutamic acid (c-PGA-MA) and thiol end-functionalized 4-arm poly (ethylene glycol) (4-arm PEG-SH) hydrogel	_	yes	_	Clinical field (for the subcutaneous delivery of trastuzumab to treat breast cancer)	[103]
Cellulose nanocrystals (CNCs) and polysilsesquioxane (PSS) aerogels with a porous hybrid structure	_	yes	_	Biomedical area (Absorbents)	[104]
Acrylic Acid/Gelatin Hydrogels	_	yes	_	Study of the effect of pH and composition on swelling and drug release (pheniramine maleate used for allergy treatment was loaded as model drug)	[105]
Scallop myosin with 1-ethyl-3-(3-dimethylaminoprolyl) carbodiimide hydrochloride (EDC), glutaraldehyde (GA), or transglutaminase (TG) gels	_	yes	_	Study of the effects of cross-linking on enzyme activity of myosin and of morphological features of myosin gel on the actins movement	[106]
Photoinduced cross-linked porcine skin gelatin with bi- and trifunctional tetrazoles	_	_	yes	Applications of polyfunctional tetrazoles in photoinduced cross-linking of biological polymers	[107]
collagen poly(vinyl pyrrolidone) (PVP)-poly(ethylene oxide) (PEO) cross-linked by e-beam irradiation in an aqueous polymeric solution	_	_	yes	Development of a new class of superabsorbent hydrogels	[108]
Polymeric gels (cream) with Glucantime (Sb V) and gel (cream) with silver nanoparticles	_	_	yes	Biomedical for alternative treatment of cutaneous Leishmaniasis	[109]
Polyvinyl alcohol (PVA) cross-linked with N,N′-methylene bis-acrylamide for the synthesis of branched polymer Dextran-graft Polyacrylaamide (D-g-PAA)	_	_	yes	Comparative study of thermal behaviour of the hydrogels for their further use in medicine	[110]
Sodium alginate microgels modified by the partial grafting of phenol groups on the backbone, in the presence of the Ru(II) catalyst complex	_	yes	yes	Applications in biology	[111]
Gelatin hydrogels cross-linked by γ -ray irradiation using ^60^Co	_	_	yes	Absorption of cationic dyes and their controlled release	[112]

**Table 3:** Most recent biomedical applications of polymer hydrogels.

Application	Polymer Hydrogel	Description	Reference
Drug delivery	oxidized succinoglycan (OSG) and a poly (N-isopropyl acrylamide-co-acrylamide) [P(NIPAM-AM)] copolymer.	stimuli-responsive drug delivery systems	[176]
	vinyl alginate (SA), acrylamide, and the hydrophobic molecule N,N′-(disulfanediylbis(4,1-phenylene))diacrylamide (SPDAAm)	controlled release of poorly water-soluble molecules	[177]
	lidocaine (LID) loaded with carboxymethyl chitosan (CCS) cross-linked with sodium alginate (SA) hydrogels	local anesthetic effect	[178]
	nano-polydopamine-reinforced hemicellulose-based hydrogels	next generation of flexible materials proper for health monitoring and self-administration	[179]
Wound healing	protocatechuic acid (PA)-mediated carboxylated chitosan (CCS) conjugated with oxidized hyaluronic acid (OHA)	antibacterial properties against common pathogens	[180]
	chitin/PEGDE-tannic acid (CPT) hydrogels	good antibacterial, antioxidant, and hemostatic activities	[181]
	incorporated gold nanorods and Ca^2+^ into polyacrylic acid and polyvinyl alcohol	the obtained hydrogel dressing could remove the wound bacterial biofilm and promote infected wound healing in vivo	[182]
Cancer treatment	bio-printed polyethylene glycol-derived hydrogels (PEG), functionalized with adhesion peptides (RGD, GFOGER and DYIGSR) and gelatin-derived and thiolated-gelatin crosslinked with PEG-4MAL)	therapy assessment in patient-derived breast cancer organoids	[183]
	cross-linked chitosan-dialdehyde guar gum Schiff-base hydrogels	chemotherapy and pain relief in CRC therapy	[123]
Biosensing	ureido pyrimidinone/tyramine (Upy/Tyr) difunctionalization of gelatin	a bidirectional neural interface for both neural recording and therapeutic electrostimulation	[184]

## Data Availability

Not applicable.
